# Genetic variability, stability and heritability for quality and yield characteristics in provitamin A cassava varieties

**DOI:** 10.1007/s10681-020-2562-7

**Published:** 2020-01-25

**Authors:** Bright Boakye Peprah, Elizabeth Parkes, Joseph Manu-Aduening, Peter Kulakow, Angeline van Biljon, Maryke Labuschagne

**Affiliations:** 1 CSIR-Crops Research Institute, Kumasi, Ghana; 2 International Institute of Tropical Agriculture, Ibadan, Nigeria; 3 Department of Plant Sciences, University of the Free State, Bloemfontein, South Africa

**Keywords:** Provitamin A cassava, Heritability, Stability, Genetic advance

## Abstract

Cassava is widely consumed in many areas of Africa, including Ghana, and is a major part of most household diets. These areas are characterized by rampant malnutrition, because the tuberous roots are low in nutritional value. Provitamin A biofortified cassava varieties have been developed by the International Institute for Tropical Agriculture, but adoption of these varieties in Ghana will largely depend on their agronomic performance, including fresh root yield, dry matter content, resistance to major pests and diseases, mealiness, starch content and the stability of these traits. Eight provitamin A varieties with two white checks were planted in three environments for two seasons to determine stability and variability among the varieties for important traits. There were significant variations in performance between varieties and between environments for cassava mosaic disease, root number, fresh root yield and starch content. High broad-sense heritability and genetic advance were observed in all traits, except for storage root number, and could be exploited through improvement programs. This study identified the best performing enhanced provitamin A varieties for traits that are key drivers of variety adoption in Ghana. In view of this, some varieties can be recommended for varietal release after on-farm testing. The study also showed the possibility of tapping heterosis after careful selection of parents.

## Introduction

The populations of underdeveloped and developing countries often suffer undernourishment and “hidden hunger” as a result of micronutrient deficiencies (Maroya et al. 2010). Areas in Africa, including Ghana, where cassava is widely consumed, are characterized by rampant malnutrition because the tuberous roots are low in nutrients such as vitamin A (Ssemakula and Dixon 2007). It is for this reason that the development of nutrient dense cassava cultivars needs much more attention to eliminate the ramifications of malnutrition among the poor in an inexpensive and sustainable way. Vitamin A deficiency constitutes a public health problem and affects mainly children and women. Recently, different programs such as HarvestPlus, involving a global alliance of research institutions, initiated the development of micronutrient-dense staple crops (Bouis et al. 2011; Dwivedi et al. 2012). Among these initiatives is the development of biofortified cassava clones with high provitamin A carotenoid content (PVAC) in the roots.

Adoption of biofortified cassava varieties in Ghana will largely depend on their agronomic performance, including storage fresh root yield, dry matter content (DMC), resistance to major pests and diseases, starch content and the stability of these traits over time and space. DMC influences texture after boiling, and is also a key parameter in the production of gari (a popular form of cassava consumption in Ghana). According to Ceballos et al. (2017), there is no negative relationship between carotenoids and DMC; thus, making it possible to identify varieties with high PVAC and acceptable levels of DMC.

Genotype by environment interaction (GEI) is the result of inconsistent performance of varieties across environments. The expression of genes that control key agronomic traits in cassava is influenced by both abiotic and biotic stresses, which results in GEI (Kang 2002). Breeders face the GEI challenge by evaluating genotypes in several environments to ensure that they have good and stable performance (Acquaah 2012). Several statistical models have been developed to interpret GEI data to understand stability. Scientists have highlighted weakness and strengths of these models, which includes commonly used ones like additive main effects and multiplicative interaction (AMMI) and genotype and genotype by environment interaction (GGE) biplots. Several studies on cassava have used AMMI for assessment of GEI effects for traits, storage root yield (Kvitschal et al. 2006; Aina et al. 2007), carotenoid and dry matter content (Maroya et al. 2010; Esuma et al. 2016) and early bulking of storage roots (Agyeman et al. 2015). The AMMI model was reported to capture a large portion of the GE sum of squares and uniquely separates main and interaction effects as required for most agricultural research purposes (Gauch 2006). Yet, the AMMI biplot does not have the most important feature of a true biplot, namely the inner-product property and this biplot does not display the discriminating ability and representativeness view of a biplot, which is effective in evaluating test environments. Hence, the GGE biplot has been proposed to effectively identify the best-performing genotypes across environments, identify the best genotypes for mega-environment delineation, whereby specific genotypes can be recommended for specific mega-environments and evaluate the yield and stability of genotypes (Yan and Kang 2003; Yan and Tinker 2006).

This study, was designed to evaluate yellow cassava clones across locations for DMC, cassava mosaic disease severity (CMDS), cassava green mite severity (CGMS), starch content, yield and its related characteristics; to determine the magnitude of genotype, environment, and GEI effects on these traits, and to identify stable and high performing clones for DMC and fresh root yield using GGE biplots.

## Materials and methods

### Varieties, experimental sites and design

Ten varieties were evaluated of which eight were selected from sets of yellow-fleshed clones previously acquired from the International Institute of Tropical Agriculture (IITA) and the other two varieties were white-fleshed landraces obtained from farmer fields in Ghana (Table 1). Trials were conducted over two seasons, May 2015–May 2016 and June 2016–June 2017 at three locations situated in different agroecological zones. Fumesua (forest), Ejura (forest transition) and Kokroko (transition). Each planting season was considered an environment, giving a total of six environments. Temperature and rainfall data were recorded during the experimentation period as well as soil nutrient profile of the fields prior to planting the trials (Table 2). Trials were laid out in a randomized complete block design with three replications, each consisting of four rows of seven plants, giving a plot size of 28 plants. Planting was done at a spacing of 1 × 1 m. To increase chances of sprouting and uniform plant establishment, all stakes used for planting were generated from the middle portions of mature stems. Replications were separated by 2 m alleys. Weeding was done when necessary and experiments were entirely rain fed.

**Table 1:** Provitamin A and white cassava genotypes used for the study

Genotype	Code	Status	Source	Pulp color
IBA090090	G1	Improved	IITA	Yellow
IBA090151	G2	Improved	IITA	Yellow
IBA070557	G3	Improved	IITA	Yellow
IBA085392	G4	Improved	IITA	Yellow
Local	G5	Landrace	Farmer	White
IBA083774	G6	Improved	IITA	Yellow
IBA070593	G7	Improved	IITA	Yellow
IBA070539	G8	Improved	IITA	Yellow
UCC	G9	Released	CSIR-CRI	White
IBA083724	G10	Improved	IITA	Yellow

**Table 2:** Characteristics of the six trial environments

Parameter	Season 1 (May 2015–May 2016)			Season 2 (June 2016–June 2017)		
	Edubiase	Kokroko	Pokuase	Edubiase	Kokroko	Pokuase
	(Env1)	(Env2)	(Env3)	(Env4)	(Env5)	(Env6)
pH	5.3	6.2	7.1	5.6	5.7	6.9
OM (%)	2.0	0.9	2.2	2.4	0.4	2.0
N (%)	0.3	0.03	0.1	0.1	0.1	0.2
P (ppm)	3.4	3.1	5.8	3.9	4.6	4.7
Ca (ppm)	3.0	1.8	5.1	3.1	2.3	5.8
Mg (ppm)	2.3	1.2	1.7	1.1	2.1	1.4
K (ppm)	0.8	0.6	0.1	0.1	3.2	0.1
Zn (ppm)	13.9	1.5	34.1	13.1	1.8	44.0
B (ppm)	0.4	0.6	0.2	0.3	0.8	0.2
Cu (ppm)	25.9	0.9	44.0	24.0	0.7	54.0
Fe (ppm)	17,365.6	3775.6	5939.38	18,365.6	3752.6	6139.28
Mn (ppm)	1618.63	470.98	1367.72	1508.63	533.72	1478.71
Rainfall (mm)	2100.0	892.9	1072.4	2350	1160.9	1420.0
Min T (°C)	21.7	32.0	30.2	21.3	32	31.0
Max T (°C)	29.1	35.0	35.8	31.1	34	34.8
Latitude	4° 40′ 0′′ N	7° 39′ 1.57′′ N	5° 42′ 0′′ N			
Longitude	1° 38′ 0′′ W	1° 56′ 56.48′′ W	0° 16′ 36′′ W			
Altitude	136.1	482.1	45.7			

*OM* organic matter content,* MinT* minimum temperature,* MaxT* maximum temperature

### Data collection

The varieties were evaluated at monthly intervals, starting at 1 month after planting (MAP) to 9 MAP, for their reaction to CMDS and CGMS. Damage symptoms were scored on a scale of 1–5, where 1 = no symptoms and 5 = very severe symptoms (IITA 1990). Only the score of the most severely affected plants were recorded in a plot. For each trial, total carotenoid content (TCC), DMC, fresh root weight (FRW) and harvest index (HI) were measured at 12 MAP. The inner two rows of each experimental plot constituted a net plot of 10 plants for measurement of the traits. Biomass from harvested plants was bulked to estimate yield components by separately weighing the fresh roots weight (FRW) and foliage (FSW). HI was computed from the measure of FRW and FSW as:

$${\text{HI}} = {\text{FRW}}/\left( {{\text{FRW}} + {\text{FSW}}} \right)$$

Root samples from each plot (5 kg) were weighed in air (Wa) using a balance after cleaning the soil and other debris from the roots. The root samples were again weighed in water (Ww). The same container was used to weigh the sample in both air and water. Specific gravity was calculated as:

$${\text{X}} = {\text{Wa}}/\left( {{\text{Wa}}{-}{\text{Ww}}} \right)$$

Dry matter and starch content were calculated using the following formulas:

$$\begin{aligned} {\text{DMC}} & = 158.3*{\text{specific}}\;{\text{gravity}} \\ &\quad - 142\left( {{\text{Kawano}}\;{\text{et}}\;{\text{al}} .\; 1 9 8 7} \right) \\ {\text{Starch}}\;{\text{content}} & = (210.8*{\text{specific}}\;{\text{gravity}}) \\ &\quad - 213.4\left( {{\text{Howeler}}\; 2 0 1 4} \right). \\ \end{aligned}$$

The mealiness was measured by taking a small portion of the boiled sample and pressing it between the thumb and the index finger. When it is soft and can form a sticky paste, it is considered mealy and suitable for ‘ampesi’ (that is boiled and eaten) or for ‘fufu’. On the other hand, the hard and difficult to press root will not form a sticky paste and is considered non-mealy. However, non-mealy genotypes can be used for cassava dough ‘agbelima’, or dried for ‘konkonte’, cassava chips or processed into gari. Components include: mealiness on a scale of 1–4 (1 = non-mealy 2 = mealy, 3 = very mealy and 4 = excellent) (Parkes 2011).

The vigor was measured 3 months after planting in terms of how the plants have germinated. The scale for measurement was 1–4 (1 = very poor, 2 = poor, 3 = good, 4 = very good) (Diniz and de Oliveira 2019).

TCC was measured following the method of Rodriquez-Amaya and Kimura (2004). Fresh cassava roots of three different sizes; small, medium and large were washed with tap water to remove dirt and debris, allowed to dry and then peeled. The peeled roots were washed with deionized water to avoid contamination and dried with tissue under a subdued light to protect carotenes. Root samples and extracts were protected from the light as much as possible. Roots were cut longitudinally in half and then the two halves were cut again longitudinally into quarters. Each quarter would include, therefore, tissue from the periphery, mid-parenchyma and core of the root, as well as proximal, central and distal sections (Chávez et al. 2008). The two quarters of each root were then ground and mixed for a uniform sample. The sample was then packaged into aluminum foil and placed into a whirl pack and labeled. Ten gram of the test sample was transferred into a clean dried mortar, and about 3 g of Celite was added to the test sample to ease maceration of the cassava tissues as well as filtration. Cold acetone (50 ml) was first added in the mortar. The mixture was crushed with a pestle until fine and then filtered. Extraction was repeated three times with cold acetone to ensure complete extraction. The extract was filtered using a Buchner funnel with 90 mm filter paper and rinsed with cold acetone.

The combined extract was transferred into a separation funnel with 5 ml distilled water and 20 ml petroleum ether. Deionized water (500 ml) was dispensed through the walls of the separation funnel to wash the acetone. Brine solution was added to break any emulsion formed in the ether extract. The petroleum ether extract containing the carotenoids was partitioned in the upper layer in the separating funnel, and the aqueous layer was gradually discarded. The extract was then transferred gradually into a 25 ml volumetric flask using a small funnel with sodium sulfate on top of cotton wool to dry any excess water. Petroleum ether was added to the extract in the volumetric flask and transferred into a 30 ml glass bottle.

Aliquots of the extracts were transferred into a Cuvette and was read using an UV–Vis spectrophotometer at wavelength of 450 nm from which absorbance readings was obtained and TCC (µg g^−1^) calculated as:

$${\text{TC}} = [ {{\text{A}} \times {\text{volume}}\left( {\text{ml}} \right) \times 10^{4} } ]/[ {{\text{A}}_{{1\,{\text{cm}}}}^{1\% } \times {\text{sample weight}}\left( {\text{g}} \right)} ]$$

where A = absorbance; volume = total volume of extract 25 ml, A_1cm_^1%^ = absorption coefficient of β-carotene in PE (2592).

All procedures for carotenoid extraction and measurement were performed in subdued light and samples were analyzed within 24 h of harvesting. TCC was measured only in 1 year for all the locations without replication (the first replication was sampled at each location) to confirm status of genotypes as provitamin A enriched.

### Data analysis

Data were subjected to analysis of variance (ANOVA) and AMMI analysis for fresh root yield, dry matter content, storage root number and starch of ten cassava plants obtained per plot across environments using Genstat software Release 17.0 (2011). Genetic effects were considered fixed, and location and season effects random. The GGE biplot method outlined by Yan (2002) was used to display the G and GE interaction patterns in the data in a biplot. The which-won-where pattern, which is an intrinsic property of the GGE biplot rendered by the inner-product property of the cassava genotype environment data set, was also visually presented. In addition, the GGE biplot was used to identify high yielding and adapted cassava varieties as well as suitable test environments.

Stable varieties for each environment were selected from AMMI analysis and principal component axes (PCA) were extracted and statistically tested by the Gollob (1968) *F* test procedure (Vargas and Crossa 2000). Phenotypic correlation coefficients and PCA and its biplot were analysed using Genstat software Release 17.0. Traits component and magnitude of variation responsiveness to selection were calculated according to Okwuagwu et al. (2008). Expected genetic advance of the mean for each trait was calculated according to Allard (1960). Genotypic and phenotypic variances were calculated according to Obilana and Fakorede (1981).

## Results

FRY ranged from 18.99 to 32.67 t ha^−1^ with a mean of 23.43 t ha^−1^ (Table 3). Genotype IBA083774 had the highest yield of 32.67 t ha^−1^, while the lowest value of 18.99 t ha^−1^ was recorded by IBA085392. DMC ranged from 23.19 to 30.26% with a mean of 27.38%. The local cultivar recorded the highest (30.25%) DMC, followed by IBA083774 (29.39%) and IBA085392 recorded the lowest value (23.19%). CMDS scores ranged from 1.0 to 2.17 with a mean of 1.15. All the yellow-fleshed varieties had a severity score of 1.0 with the exception of IBA070593 (1.17). The local cultivar recorded the highest severity score (2.17) to CMDS. All the elite cassava genotypes from IITA recorded higher TCC values than the local check used. Genotype IBA083774 with the highest root yield recorded the lowest TCC values among the IITA materials and the local check with the highest DMC recorded the lowest TCC value.

**Table 3:** Mean of ten traits measured in 10 genotypes across six environments in Ghana

Genotype	FRY	RTN	TWT	DMC	Starch	HI	CMDS	CGMS	Mealy	TCC
IBA090090	19.69	41.17	35.79	26.95	13.24	0.45	1.00	1.27	1.94	10.37
Local	22.69	45.67	38.64	30.26	15.68	0.44	2.17	1.67	3.33	0.78
IBA090151	25.03	56.06	39.04	27.62	13.72	0.34	1.00	1.05	1.67	12.73
IBA070557	22.58	43.83	34.42	28.76	14.52	0.39	1.00	1.11	1.89	7.78
IBA085392	18.99	42.56	40.62	23.19	10.59	0.31	1.00	1.16	1.50	11.74
IBA083724	24.62	56.67	30.23	29.16	14.81	0.36	1.00	1.12	1.89	6.58
IBA083774	32.67	56.50	39.97	29.39	14.97	0.46	1.00	1.50	1.06	3.12
IBA070593	19.19	35.44	33.72	26.37	12.83	0.35	1.17	1.61	1.94	16.00
IBA070539	22.48	41.11	23.27	23.89	11.08	0.47	1.00	1.38	1.66	13.79
UCC	26.47	44.06	24.84	28.23	14.15	0.51	1.17	1.33	2.50	3.13
Grand mean	23.43	46.31	34.04	27.38	13.54	0.41	1.15	1.33	1.93	8.60
S.e.d	6.15	10.82	7.89	1.47	2.06	0.05	0.18	0.47	0.76	1.56
CV %	32.10	28.60	28.40	6.60	9.40	15.50	19.60	43.20	48.20	59.70

*FRY* fresh root weight (t ha^−1^),* RTN* storage root number (t ha^−1^),* TWT* total biomass (t ha^−1^),* DMC* dry matter content (%),* HI* harvest index,* CMDS* cassava mosaic disease severity,* CGMS* cassava green mite severity,* TCC* total carotenoid content (µg g^−1^)

In the combined ANOVA (Table 4) the main effects (genotype, location and year) were highly significant (*P* < 0.001) for all traits evaluated except CMDS. Combined AMMI ANOVA (Table 5) showed that genotype, environment and GEI effects were highly significant (*P* < 0.001) for CMDS, DMC, FRY, RTN and starch. IPCA1 mean squares were highly significant (*P* < 0.001) for all traits except FRY, which was significant at *P* < 0.01. IPCA1 and IPCA2 accounted for more than 70% of the total variation observed in GEI, which was confirmed by the significant (*P* < 0.001) GEI effects for all traits.

**Table 4:** Analysis of variance and contribution of main effects to variation for measured characteristics across three environments in two growing seasons

Characteristic	Source	*df*	SS	MS	% of total SS
CMDS	Genotype	9	21.45	2.38***	65.10
	Location	2	0.23	0.12 ns	0.70
	Year	1	0.27	0.27*	0.82
	Gen.loc	18	1.43	0.08 ns	4.34
	Gen.year	9	1.67	0.19***	5.07
	Loc.year	2	0.21	0.11 ns	0.64
	Gen.loc.year	18	1.68	0.09*	5.10
	Error	118	6.00	0.05	
DMC	Genotype	9	883.66	98.19***	39.96
	Location	2	245.34	122.67***	11.10
	Year	1	189.11	189.11***	8.55
	Gen.loc	18	191.27	85.75***	8.65
	Gen.year	9	57.47	6.39*	2.60
	Loc.year	2	171.50	85.75***	7.76
	Gen.loc.year	18	86.49	4.80 ns	3.91
	Error	118	380.27	3.22	
Starch	Genotype	9	443.13	49.24***	39.96
	Location	2	123.03	61.52***	11.10
	Year	1	94.83	94.83***	8.55
	Gen.loc	18	95.91	5.33***	8.65
	Gen.year	9	28.82	3.20*	2.60
	Loc.year	2	86.01	43.00**	7.76
	Gen.loc.year	18	43.38	2.41 ns	3.91
	Error	118	190.70	1.62	
FRY	Genotype	9	2758.90	306.40***	12.02
	Location	2	4413.48	2206.74***	19.22
	Year	1	3029.08	3029.08***	13.19
	Gen.loc	18	1499.40	83.30 ns	6.53
	Gen.year	9	671.50	74.61 ns	2.92
	Loc.year	2	2189.76	1094.88***	9.54
	Gen.loc.year	18	1648.67	91.59*	7.18
	Error	118	6685.96	56.66	
RTN	Genotype	9	9060.10	1006.70***	16.39
	Location	2	3390.30	1695.20***	6.13
	Year	1	1566.50	1566.50***	2.83
	Gen.loc	18	10,419.90	578.90***	18.85
	Gen.year	9	3031.20	336.80 ns	5.48
	Loc.year	2	1073.10	536.50 ns	1.94
	Gen.loc.year	18	5854.50	325.20*	0.11
	Error	118	20,725.90	175.60	

*CMDS* cassava mosaic disease severity,* DMC* dry matter content,* FRY* fresh root weight,* RTN* storage root number

*P ≤ 0.05; ** *P* ≤ 0.01; ***P ≤ 0.001

**Table 5:** AMMI analysis of variance for five of the measured characteristics

Source	*df*	CMDS	DMC	RTN	FRY	Starch
Genotype	9	2.38***	98.18***	1006.7***	306.5***	49.24***
Environment	5	0.14***	121.19***	1206.0***	926.5***	60.77***
GEI	45	0.11***	7.45***	429.0***	84.9***	3.74***
IPCA1	13	0.28***	15.83***	770.3***	127.5**	7.94***
IPCA2	11	0.07 ns	3.72 ns	396.8***	89.4*	1.87 ns
Residual	21	0.02	4.210	234.60	56.10	2.11
% GEI due to IPCA1		76.29	61.40	51.87	43.40	61.39
% GEI due to IPCA2		15.89	12.23	22.61	25.76	12.20

*CMDS* cassava mosaic disease severity,* DMC* dry matter content,* RTN* storage root number,* FRY* fresh root weight,* GEI* genotype by environment interaction,* IPCA* interaction principal component axis

*P ≤ 0.05; ** *P* ≤ 0.01; ***P ≤ 0.001

RTN and TWT, FRY and TWT, TWT and vigor, HI and RTN, HI and FRY, CMDS and mealiness, RTN and FRY and CGMS and HI were highly significantly positively correlated. TWT and HI and vigor and HI showed significant negative correlations (Table 6).

**Table 6:** Phenotypic correlations coefficients for 10 traits measured on 10 cassava genotypes across six environments in Ghana

Traits	CGMS	CMDS	DMC	HI	Mealy	RTN	FRY	TWT
CMDS	0.13							
DMC	0.03	0.18*						
HI	0.33	0.05	0.08					
Mealy	− 0.01	0.29***	− 0.001	− 0.01				
RTN	0.20**	0.03	0.16*	0.45***	− 0.07			
FRY	0.19*	− 0.05	0.07	0.62***	− 0.18*	0.69***		
TWT	− 0.18*	− 0.13*	− 0.02	− 0.43***	− 0.13	0.30***	0.36***	
Vigor	− 0.12	− 0.04*	0.14	− 0.22**	− 0.03	0.07	0.05	0.33**

*CGMS* cassava green mite severity,* CMDS* cassava mosaic disease severity,* DMC* dry matter content,* HI* harvest index,* RTN* storage root number,* FRY* fresh root weight,* TWT* total biomass

*P ≤ 0.05; **P ≤ 0.01; ***P ≤ 0.001

The magnitude of the phenotypic coefficient of variation (PCV) was higher than their corresponding genotypic coefficient of variation (GCV) for all the traits studied. The PCV ranged between 8.55% and 26.09%, with CMDS showing the highest value, followed by TWT and with DMC recording the lowest value. Heritability was generally high for all characteristics and varied from 41.34% for RTN to 88.89% for CMDS (Table 7).

**Table 7:** Coefficients of variation, heritability and genetic advance for five traits of 10 cassava genotypes planted in six environments

Traits	Genetic parameters				
	Mean	GCV	PCV (%)	H_b_^2^	GAs
FRY	25.33	15.58	17.63	78.39	26.27
RTN	46.31	10.39	16.15	41.34	13.76
TWT	34.04	14.04	18.10	60.10	22.40
Starch	13.55	11.44	13.14	75.95	20.55
DMC	27.38	8.00	8.55	87.55	15.41
CMDS	1.15	24.35	26.09	88.89	47.77

*GVC* genotypic coefficient of variation (%),* PCV* phenotypic coefficient of variation (%), H_b_^2^, broad-sense heritability (%),* GAs* expected genetic advance of the mean,* FRY* fresh root weight,* RTN* storage root number,* TWT* total biomass,* DMC* dry matter content,* CMDS* cassava mosaic disease severity

From the PCA (Table 8) the first three principal components (PC) had eigenvalues higher than one and accounted for 83.93% of the total variation. PC1 accounted for 40.50% variation and RTN, FRY, DMC, starch and CMDS were the principal contributors. PC2 accounted for 27.51% of the variation with TWT and vigor (positively) and CGMS and mealiness (negatively) contributing most to the variation. PC3 accounted for 15.92% of variation with FRY, mealiness, CMDS and HI being the main contributing factors.

**Table 8:** Principal component analysis of 10 quantitative traits in 10 cassava genotypes showing eigenvectors, eigenvalues, individual and cumulative percentage of variation explained by the first three PC axis

Characters	Eigenvectors		
	PC1	PC2	PC3
RTN	0.34	0.27	0.06
FRY	0.35	0.23	0.47
TWT	− 0.09	0.52	− 0.32
Mealy	0.28	− 0.33	− 0.41
DMC	0.43	0.16	− 0.14
Starch	0.43	0.16	− 0.14
Vigor	0.12	0.49	− 0.13
CMDS	0.34	− 0.27	− 0.37
CGMS	0.24	− 0.31	− 0.06
HI	0.32	− 0.19	− 0.56
Eigenvalue	4.05	2.75	1.50
Individual	40.50	27.51	15.92
Cumulative	40.50	68.01	83.93

*RTN* storage root number,* FRY* fresh root weight,* TWT* total biomass,* DMC* dry matter content,* CMDS* cassava mosaic disease severity,* CGMS* cassava green mite severity,* HI* harvest index

### GGE biplot for average dry matter content, fresh root yield, starch and stability of varieties

**Fig. 1:**
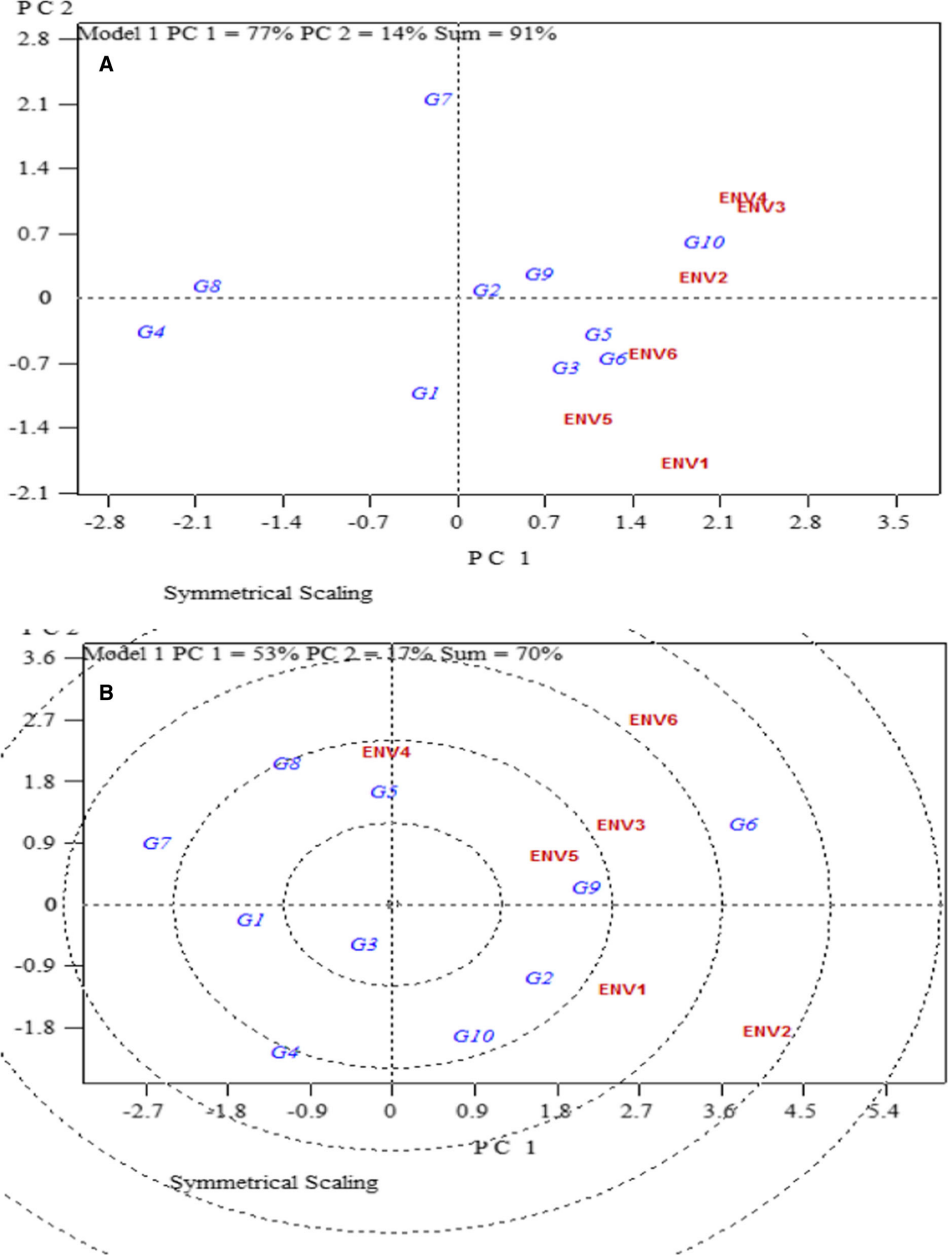
GGE biplot showing **a** dry matter content and **b** fresh root yield mean performance and stability of 10 cassava genotypes

The biplot (Fig. 1) showed that PCA1 and PCA2 explained 91% of variation for DMC. DMC was highest in genotype IBA083724 (G10), followed by IBA083774 (G6) and local (G5). IBA085392 (G4) had the lowest DMC value. Varieties IBA090151 (G2) and IBA070539 (G8) were more stable with genotype IBA070593 (G7) being the most unstable. Genotype IBA083774 (G6) had the highest mean for FRY, followed by UCC (G9), IBA090151 (G2), while IBA070593 (G7) ranked the lowest (Fig. 1). In terms of stability, varieties IBA090090 (G1) and UCC (G9) were most stable.

### The best performing genotype in each environment and mega-environments for dry matter content, fresh root yield and starch content

**Fig. 2:**
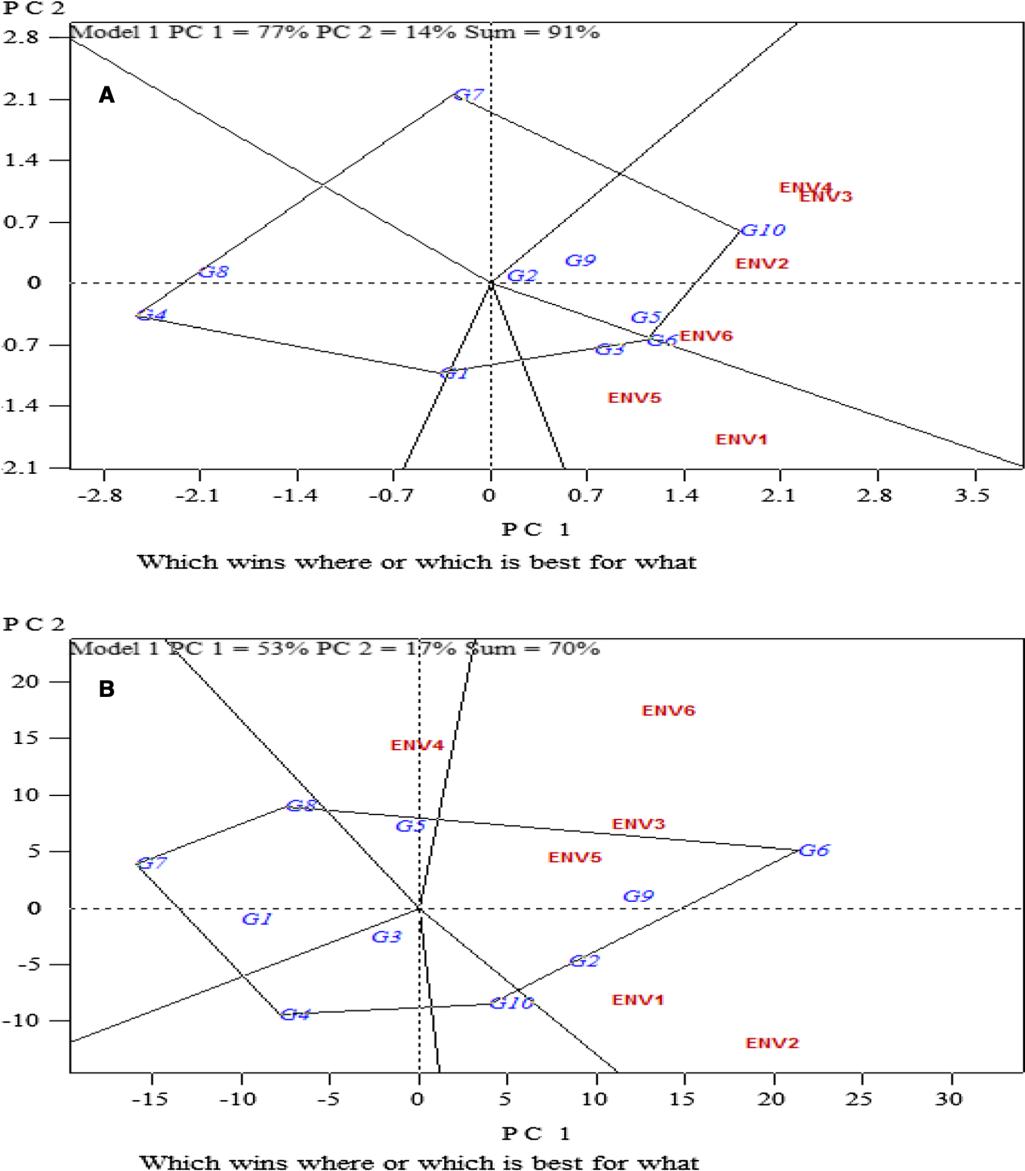
Which wins where GGE biplot for best cultivars for **a** dry matter content and **b** fresh root yield in different environments

PC1 explained 77% and PC2 14% of variation, both reflecting 91% of the DMC variation (Fig. 2). PC1 explained 53% and PC2 17% of variation in FRY, reflecting a total 70% of variation.

A convex-hull drawn on the varieties from the origin of the biplot gave five sections with IBA070593 (G7), IBA085392 (G4), IBA090090 (G1), IBA083774 (G6) and IBA083724 (G10) as vertex varieties. G10 was the best variety in three environments (Edubiase, Env 4; Pokuase, Env 3; Kokroko, Env 2 and Pokuase, Env 6) and G6 was best in two environments (Edubiase, Env 1 and Kokroko, Env 5) for DMC (Fig. 2). The biplot grouped all the environments into two groups, suggesting two mega-environments. The first mega-environment had environments Env 4, Env 3, Env 2 and Env 6 with varieties IBA083724 (G10), UCC (G9), Local (G5), IBA090151 (G2) and IBA083774 (G6) as the best performer and the second mega-environment had environments Env 5 and Env 1, with genotype IBA070557 (G3) performing best.

For FRY, IBA083774 (G6), IBA070539 (G8), IBA070593 (G7), IBA085392 (G4) and IBA083724 (G10) were the vertex varieties for the five sections of the biplot (Fig. 2). The biplot grouped all the environments into two groups, suggesting two mega-environments. The first mega-environment had environments Env 1, Env 2, Env 3, Env 5 and Env 6 with varieties UCC (G9), IBA090151 (G2) and IBA083774 (G6) as the best performers and the second mega-environment had environment Env 4, with genotype Local (G5) performing the best.

## Discussion

DMC, FRY, starch, CMDS, mealiness and RTN are key drivers for cassava variety adoption (Abdoulaye et al. 2014; Esuma et al. 2016). All the yellow-fleshed varieties in this study had higher TCC values than the local and improved check. Three of the yellow-flesh varieties (IBA090I51, IBA083774 and IBA083724) recorded higher FRY than the checks. In terms of DMC, the local variety was not statistically different from varieties IBA083774 and IBA083724, which recorded the highest FRY and lowest CMDS score.

There were significant variations in mean performance of varieties for CMDS, RTN, FRY and starch, which are some of the most important traits for consumer acceptance (Owusu and Donkor 2012), in different environments. TCC-rich cassava cultivars could be selected using on- station trial in one location and later subjected (selected cultivars) to multi- location evaluations where the focus is shifted to other important traits of cassava for variety adoption (Esuma et al. 2016).

The significant genotype effects observed for the traits studied indicated that varieties were significantly different, hence genetic improvement could be achieved through hybridization. The significant GEI (from AMMI analysis) for CMDS, DMC, RTN, FRY and starch, indicated variation in genotypic responses to different environments and this underlined the importance of the multi-environment testing of newly developed varieties.

The combined ANOVA for CMDS, DMC and starch indicated that genotype main effect accounted for 65.10%, 39.96% and 39.96% of variation, respectively. This was confirmed by the small difference between their PCV and GCV values. Selection for such traits could be fairly easy due to the close association between the genotype and the phenotype.

Cassava breeding aimed at selecting desired genotypes is linked with GCV, heritability estimates, genetic advance as percentage of the population mean and other genetic parameters for important traits (Idahosa et al. 2010). The magnitude of the heritability of the selected traits studied were generally high. Pradeepkumar et al. (2001) reported that heritability estimates together with genetic advance contribute to improved selection response. The low PCV values for DMC in this study have also been reported by other authors (Kundy et al. 2015; Ewa et al. 2017). The generally higher values of PCV than their corresponding GCV values for traits indicated the considerable role of the environment in the expression of these traits; hence the variation in the varieties are due to both genotype and the environment. The high heritability values for the measured traits indicate the presence of a larger portion of heritable variation which would aid selection. Root number with quite high heritability and low genetic advance could pose a challenge if selection is based only on this trait. Esuma et al. (2016) confirmed a strong negative correlation between DMC and TCC. Ceballos et al. (2013) reported simultaneous gains for both TCC and DMC through rapid selection. There is need in Ghana, to combine these two important traits in the breeding program. The best yellow-flesh varieties identified in this study could be the starting material for this improvement.

Correlations among traits play an important role in plant breeding by improving selection efficiency. The positive significant correlation between FRY and RTN, TWT, HI and DMC and starch, suggests that an increase in mean value of any one of these character pairs would significantly increase the mean of the other (Akinwale et al. 2010). The negative significant correlation of HI and TWT is very important in cassava breeding where the ultimate focus is on yield (storage roots) which correlates positively with HI. However, varieties must also produce prolific stems from planting material that is related to TWT. The negative correlations between CMDS and FRY, TWT and vigor confirms the potential storage root yield losses that can be caused by the disease, which was confirmed by Parkes et al. (2013). There was also a significant positive correlation between CMDS and mealiness. Landraces are more susceptible to CMDS and most landraces in Ghana are mealy.

A threshold of 15 µg g^−1^ has been set as a goal in cassava for PVAC in terms of nutritional enhancement (Njoku et al. 2011). In the current study the TCC values of the yellow varieties varied between 3.12 and 16 µg g^−1^ with five varieties having values of more than 10 µg g^−1^. These varieties would certainly have health benefits for consumers, and the other varieties to a lesser extent.

## Conclusions

This study showed the best performing TCC-rich varieties also have variation for important traits of cassava, which are key drivers of variety adoption in Ghana. In view of this, varieties IBA090151, IBA083774 and IBA083724 can be considered for varietal release after on-farm testing. The study also revealed that the yellow-fleshed varieties can be used in a hybridization scheme with the local material to combine both TCC and DMC traits with high yield in a CMDS free background.
